# Age- and cognitive load–related variability and entropy of gait: integrating coefficient of variation, median absolute deviation, and permutation entropy of spatiotemporal parameters into the Semmelweis Study gait assessment framework

**DOI:** 10.1007/s11357-026-02256-1

**Published:** 2026-05-08

**Authors:** Peter Mukli, Mihaly Muranyi, Ágnes Lipecz, Zsofia Szarvas, Tamás Csípő, Mónika Fekete, Vince Fazekas-Pongor, Anna Peterfi, Ágnes Fehér, Norbert Dosa, Csilla Kaposvári, Anna Aliquander, Wei Yi Hung, Dávid Major, Zalan Kaposzta, Attila Matiscsák, Gabriella Dörnyei, Zoltan Benyo, Roland Patai, Boglarka Csík, Rafal Gulej, Anna Ungvari, Panna T. Pónyai, Emese Győrffy, Attila Kállai, Márton Sándor, Peter Varga, Adam G. Tabak, Stefano Tarantini, Róza Ádány, Béla Merkely, Anna Csiszar, Andriy Yabluchanskiy, Zoltan Ungvari

**Affiliations:** 1 Vascular Cognitive Impairment, Neurodegeneration and Healthy Brain Aging Program, Department of Neurosurgery, University of Oklahoma Health Sciences Center, Oklahoma City, OK USA; 2 Oklahoma Center for Geroscience and Healthy Brain Aging, University of Oklahoma Health Sciences Center, Oklahoma City, OK USA; 3 International Training Program in Geroscience, Doctoral College, Health Sciences Division/Institute of Preventive Medicine and Public Health, Semmelweis University, Budapest, Hungary; 4 Doctoral College, Health Sciences Division, Semmelweis University, Budapest, Hungary; 5 Institute of Preventive Medicine and Public Health, Semmelweis University, Budapest, Hungary; 6 Fodor Center for Prevention and Healthy Aging, Semmelweis University, Budapest, Hungary; 7 Faculty of Health Sciences, Semmelweis University, Budapest, Hungary; 8 Institute of Translational Medicine, Semmelweis University, 1094 Budapest, Hungary; 9 Cerebrovascular and Neurocognitive Disorders Research Group, HUN-REN, Semmelweis University, 1094 Budapest, Hungary; 10 Institute for Translational Research, Budapest, Hungary; 11 UCL Brain Sciences, University College London, London, UK; 12 Department of Internal Medicine and Oncology, Faculty of Medicine, Semmelweis University, Budapest, Hungary; 13 Department of Health Promotion Sciences, College of Public Health, University of Oklahoma Health Sciences Center, Oklahoma City, OK USA; 14 The Peggy and Charles Stephenson Cancer Center, University of Oklahoma Health Sciences Center, Oklahoma City, OK USA; 15 Department of Public Health and Epidemiology, Faculty of Medicine, University of Debrecen, Debrecen, Hungary; 16 HUN-REN-UD Public Health Research Group, Department of Public Health and Epidemiology, Faculty of Medicine, University of Debrecen, Debrecen, Hungary; 17 National Laboratory for Health Security, Center for Epidemiology and Surveillance, Semmelweis University, Budapest, Hungary; 18 Heart and Vascular Center, Semmelweis University, Budapest, Hungary

**Keywords:** Aging, Gait variability, Cognitive–motor integration, Median absolute deviation, Coefficient of variation, Permutation entropy, Dual-task walking, Cerebrovascular aging, Semmelweis Study, Healthy longevity

## Abstract

Aging profoundly alters the neuromotor and cognitive systems that support gait control, leading to increased variability and instability that predict functional decline and dementia risk. In this pilot study, conducted to inform the design of the Semmelweis Study gait assessment pipeline, we examined how aging and cognitive load influence the magnitude and temporal organization of gait fluctuations. The Semmelweis Study is a large, prospective workplace cohort at Semmelweis University designed to identify the determinants of unhealthy aging and the mechanisms that preserve functional resilience across the life course. One hundred three adults aged 23–87 years completed single- and dual-task walking trials on a 20-foot pressure-sensitive walkway. Gait variability was quantified using the median absolute deviation (MAD) and coefficient of variation (CoV) of key spatiotemporal parameters, while permutation entropy (PE) captured the complexity of stride-to-stride dynamics. Aging was associated with progressive increases in both the variability (MAD, CoV) and changes in orderliness (PE) of gait fluctuations, particularly under dual-task conditions, suggesting a dual contribution of neuromotor degradation and compensatory recruitment of higher-order control processes. The amplification of these effects during cognitive load highlights the vulnerability of cognitive–motor integration with advancing age. By integrating robust, relative, and nonlinear variability metrics within a unified analytical framework, this study provides a multidimensional characterization of gait control and establishes sensitive indicators for detecting early functional decline. Within the translational framework of the Semmelweis Study, these quantitative gait measures—together with vascular, metabolic, and cognitive assessments—are expected to serve as informative components of a comprehensive biomarker system aimed at identifying early determinants of unhealthy brain aging and guiding preventive strategies to promote healthy longevity.

## Introduction

Europe is entering an unprecedented era of population aging [1] that is redefining the boundaries of medicine, public health, and social policy [2]. The demographic shift toward older age is not merely a numerical trend, it represents a fundamental transformation in the health needs and functional capacities of societies [1–4]. Although advances in prevention and medical care have extended life expectancy across much of Europe, this achievement is increasingly overshadowed in many countries by the premature manifestation of age-related chronic diseases [2, 5–10]. These challenges are particularly pronounced in Central and Eastern Europe, where disparities in life expectancy and healthy life years remain among the widest in the EU [5, 7, 8, 11–13]. The growing prevalence of cardiovascular, metabolic, neurodegenerative [10], and musculoskeletal disorders in these countries reflects the cumulative impact of unhealthy lifestyles [14] and environmental exposures [15], which accelerate biological aging and erode functional reserve. These conditions, often interlinked through shared vascular, inflammatory, and metabolic mechanisms, drive the process of unhealthy aging, a state characterized by accelerated physiological decline and increased morbidity and mortality of chronic non-communicable diseases. Unhealthy population aging not only diminishes quality of life but also undermines the sustainability of healthcare systems and social support networks [2]. In Hungary, where life expectancy remains below the EU average and the burden of age-associated morbidity is particularly high, the promotion of healthy aging and preservation of physical and cognitive function have become a key public health priority [16–18]. Addressing these challenges requires integrative, preventive strategies that transcend traditional disease-specific paradigms and instead focus on optimizing the biological processes of aging and extending healthspan—ensuring that longer lives are accompanied by preserved function, vitality, and independence.

In response to these challenges, Semmelweis University has launched a comprehensive strategy to promote healthy longevity, integrating research, education, and clinical innovation under the umbrella of the Fodor József Center for Prevention and Healthy Aging. At the heart of this initiative lies the Semmelweis Study [19], a prospective, university-wide workplace cohort designed to identify the determinants of unhealthy aging and the mechanisms that preserve functional resilience. Encompassing a significant proportion of the university’s more than 16,000 employees from diverse occupational and socioeconomic backgrounds, the Semmelweis Study provides an unprecedented opportunity to investigate how lifestyle, cardiovascular, metabolic, and psychosocial factors shape aging trajectories within a real-world working population. By combining epidemiological, clinical, and high-resolution physiological assessments, the program seeks to elucidate the biological and behavioral pathways that link vascular and metabolic aging to cognitive and motor function. This multidimensional approach positions the Semmelweis Study as a model for translational aging research in Central and Eastern Europe—bridging scientific discovery with public health implementation and aligning university-based innovation with national prevention priorities.

Within this multidisciplinary framework, gait analysis has emerged as a critical component of the Semmelweis Study’s functional aging assessment strategy [20]. Once considered a simple biomechanical process, gait is now recognized as a complex neurobehavioral function reflecting the integrity of cortical, subcortical, and spinal networks involved in motor control, balance, and executive function [21–26]. Accumulating evidence links subtle gait alterations to vascular brain injury, white matter microstructural damage, and early cognitive decline [21, 27–37]. As such, quantitative gait analysis serves as a window into brain health, offering a sensitive, non-invasive means of capturing early signs of neural aging [21, 28–37]. Incorporating gait assessment into population-based studies such as the Semmelweis Study enables the identification of individuals at risk for functional and cognitive impairment long before clinical symptoms emerge. The Semmelweis Study is designed as a prospective longitudinal cohort, with repeated assessments planned at 5-year intervals, enabling evaluation of temporal trajectories of gait and their relationship to cognitive and vascular aging.

Among the parameters characterizing gait, gait variability, the natural stride-to-stride fluctuation in spatiotemporal parameters, has gained increasing attention as a biomarker of neuromotor control and cognitive integrity [29, 34, 38–42]. Unlike mean gait measures, which reflect overall performance, gait variability captures the consistency and stability of locomotion, offering insight into the adaptability and precision of the underlying neural control mechanisms. Physiologically, low variability indicates robust neuromotor coordination and stable sensorimotor feedback loops, whereas elevated variability reflects increased neural noise, degraded proprioceptive integration, and impaired executive control [43, 44]. Importantly, excessive gait variability has been associated with frailty, increased fall risk, and cognitive impairment, and predicts adverse outcomes including hospitalization, loss of independence, and mortality, even after accounting for gait speed and other mean gait measures [43, 45–51].

Aging is consistently associated with increased variability in temporal and spatial gait parameters, reflecting both peripheral and central mechanisms of decline [29, 34, 38–42]. With advancing age, degradation of white matter tracts, cerebellar atrophy, and impaired cortical-subcortical connectivity contribute to noisier motor output and less stable gait patterns. The dual-task paradigm, which combines walking with a concurrent cognitive challenge, provides an additional dimension for assessing the vulnerability of gait control systems under cognitive load [29]. Older adults typically exhibit disproportionate increases in gait variability during dual-task walking, suggesting reduced cognitive-motor integration and diminished compensatory capacity. Recent work has introduced entropy-based metrics, which quantify the *complexity and temporal organization* of gait patterns [52–54]. While increased variability often signals instability, altered entropy may reflect adjustments in higher-order neural mechanisms under cognitive or motor stress. However, it remains unclear how gait variability metrics compare in their sensitivity to aging and cognitive load within a unified analytical framework.

In this pilot study, conducted to inform and optimize the gait assessment protocol for the Semmelweis Study, we focus on gait variability, quantified through the coefficient of variation (CoV), the median absolute deviation (MAD), and permutation entropy (PE) of key spatiotemporal gait parameters. These complementary measures capture distinct yet interrelated aspects of gait control: CoV reflects relative dispersion normalized to mean performance, MAD provides a robust estimate of absolute stride-to-stride fluctuation that is less sensitive to outliers or non-normal distributions, and PE quantifies the orderliness and predictability of temporal gait dynamics, offering insights into higher-order neuromotor adaptability. By systematically evaluating age- and cognitive load–related changes across these variability metrics, this pilot aims to identify the most sensitive indicators of early functional decline, refine the Semmelweis Study gait assessment protocol, and lay groundwork for future longitudinal analyses linking gait variability and complexity of stride-to-stride fluctuations to vascular, metabolic, and neurocognitive outcomes.

## Materials and methods

### Study participants

This cross-sectional study included 103 community-dwelling adults aged 23 to 87 years (*45 males*). Participants were categorized into two age groups based on established cut-off values. The Younger Adults (YA) group (< 65 years, *n = 62*; *27 males*, *35 females*) encompassed both young and middle-aged adults, with an internal subdivision at 45 years. The Older Adults (OA) group (≥ 65 years, *n* = *41*; *18 males*,* 23 females*) represented the elderly population.

All participants underwent medical screening prior to enrollment. Inclusion criteria required reading and writing proficiency, adequate hearing and vision for the assessments, and the ability to provide informed consent and able to walk unassisted. Exclusion criteria consisted of the following: significant cardiac disease (e.g., heart failure), chest pain in the last 6 months, stage-2 high blood pressure not controlled by medication (> 160/100 mm Hg), history of diabetes mellitus not controlled by medication, multiple sclerosis, active cancer, anxiety disorder, patients with established vascular disease (such as Raynaud’s syndrome), pregnancy or breast-feeding. For this cross-sectional analysis, we excluded participants with Parkinson’s disease since marked gait alteration is a hallmark of this condition. The cohort included individuals with common, controlled cardiovascular risk factors, reflecting a real-world population. The influence of these factors on gait variability will be examined in future analyses.

To minimize confounding factors, participants were asked to abstain from caffeine for at least six hours before testing and to obtain a minimum of seven hours of sleep on the preceding night. Written informed consent was obtained from all participants before participation. The study protocol was reviewed and approved by the institutional review boards of the participating institutions and the Hungarian Medical Research Council (Semmelweis University: 53981–2/2023/809; OUHSC IRB: No. 8129 and 9555).

### Measurement protocol

Gait data were collected using a ~ 6-m pressure-sensitive electronic walkway (ProtoKinetics Zeno™ Walkway Gait Analysis System, Havertown, PA, USA), which records both spatial (distance-related) and temporal (time-related) gait parameters with high precision. Participants walked at their natural pace, wearing their regular shoes, and were free from external constraints. Each participant completed two conditions:

1. Single-task walking: participants were instructed to complete five laps (ten passes) on the gait mat in their natural pace, with turns at each end.
2. Dual-task walking: to introduce cognitive load, participants performed serial subtraction (subtracting 7 repeatedly from 500) while walking another five laps. They were instructed to verbalize each number and continue even if an earlier error occurred, ensuring sustained cognitive engagement. Participants were instructed to continuously perform serial subtraction to maintain cognitive engagement; however, detailed quantitative cognitive performance metrics were not analyzed in this pilot study.

This dual-task paradigm was used to probe cognitive–motor interference and attentional resource allocation during walking—factors that typically reveal age-related limitations in executive function and motor control.

### Assessment of gait patterns

Gait patterns were characterized using a comprehensive set of spatiotemporal parameters (Table 1) automatically extracted from the ProtoKinetics Movement Analysis Software (PKMAS). A comprehensive characterization of gait patterns includes spatial and temporal profiles as well as key parameters reflecting balance control across different phases of the gait cycle.

**Table 1:** Parameters of gait pattern captured by the pressure-sensitive gait mat

Gait parameter name (abbreviation)	Definition (measurement unit)
*Gait speed*	The covered distance during a given walking trial divided by time
*Cadence*	The number of steps during a given walking trial divided by time
*Step Time*	It refers to the duration of a single step, measured as the time interval between the initial contact of one foot and the initial contact of the opposite foot. (sec)
*Stride Time*	A key parameter capturing the duration of gait cycle: the period of time from first contact of one foot, to the following first contact of the same foot (sec)
*Step Length*	Step Length is the distance between corresponding successive heel points of opposite feet, measured parallel to the direction of progression for the ipsilateral stride (cm)
*Stride Length*	The distance from the heel of one foot to the following heel of the same foot (cm)
*Stride Width*	Stride Width is the perpendicular distance between the line connecting the two ipsilateral foot heel contacts (stride) with the contralateral heel contact between those events (cm)
*Stance*	The stance phase begins when the foot first touches the ground and ends when the same foot leaves the ground. This measure is expressed as a percentage of gait cycle time spent in the stance phase and averaged between left and right foot (%)
*Swing*	The period of time when the foot is not in contact with the ground. It is presented as a percentage of the gait cycle time (%)
*Single Support*	The percentage of gait cycle time when only the current foot is in contact with the ground (%)
*Total Double Support*	The percentage of gait cycle time when both feet are in contact with the ground during stance phase (%)
*Integrated pressure*	The area under the footfall pressure curve during ground contact for the given footfall
*Stance Center of Pressure distance*	It represents the center of pressure (COP) start to end distance as a percent of the maximum foot length
*Single Support Center of Pressure distance*	It represents the COP start to end distance in single support as a percent of the maximum foot length

Temporal parameters include gait speed, cadence, step time, and stride time, which provide insights into the dynamics; while spatial parameters such as step length, stride length, and stride width describe the overall geometry of locomotion. The relative durations of gait cycle phases are assessed through stance, single support, and dual support times, offering critical information about weight distribution and stability during walking. Additionally, parameters reflecting balance control include integrated pressure, stance center of pressure (CoP) distance, and single support center of pressure distance. These measures provide insights into postural stability and neuromotor control, offering a more detailed evaluation of balance across different gait phases.

The reliability of the extracted data was verified through visual inspection of step sequences and consistency across trials. Artefactual recordings that typically arising from incomplete steps, irregular turning or acceleration phases were excluded prior to calculation of gait outcome measures. This standardized approach ensured that all derived parameters reflected steady-state walking and were suitable for subsequent variability and complexity analyses (CoV, MAD, and PE).

### Data analysis

After data cleaning, individual-level analyses were conducted by calculating the arithmetic mean for each gait parameter listed in Table 1. To characterize step-to-step variability and signal complexity, three complementary measures were derived: the Median Absolute Deviation (MAD) and Coefficient of Variation (CoV) as indicators of *gait variability*, and Permutation Entropy (PE) as a measure of *gait orderliness*.

The MAD is a robust measure of statistical dispersion that quantifies the typical absolute distance of individual observations from the dataset’s median. Unlike the standard deviation, which is sensitive to outliers and assumes normally distributed data, MAD provides a resistant estimate of variability that remains stable even when the distribution is skewed or contains extreme values. In the context of gait analysis, MAD captures the *magnitude* of stride-to-stride fluctuations within each parameter, offering a reliable index of gait consistency. For consistency across participants, MAD was calculated only for the right leg for each gait parameter, providing a representative index of intra-limb variability. The right leg was selected for analysis to ensure consistency and avoid redundancy, as preliminary analyses confirmed no systematic differences between left and right limbs. Raw gait data were first separated by leg, and MAD values for the right leg were computed using the following formula:

1 $$MAD\;\left(x\right)=median\;\left(\left|x-median\right|\right)$$

This approach ensures that variability estimates reflect steady-state walking behavior and are minimally influenced by outliers or transient fluctuations.

The coefficient of variation (CoV) is a normalized measure of relative variability that expresses the standard deviation as a proportion of the mean. It provides a dimensionless index of dispersion, allowing direct comparison of variability across parameters with different units or magnitudes. In gait analysis, CoV captures the *consistency and stability* of repetitive motor patterns—quantifying how much a given gait parameter fluctuates relative to its average value. A lower CoV indicates highly regular, stable gait cycles typically observed in younger or neurologically healthy individuals, whereas a higher CoV reflects increased stride-to-stride irregularity, often associated with aging, neuromotor noise, or impaired balance control. CoV was calculated for each gait parameter according to Eq. (2):

2 $$CoV=\frac{SD}{Mean}$$

By normalizing variability to the mean, CoV facilitates meaningful comparisons both within and between individuals, across different gait parameters, and between single- and dual-task conditions.

In this study, we characterized the predictability of stride-to-stride sequences by calculating PE, an entropy measure that is applicable for shorter gait time series with *N* < 200 steps [55], where *N* is the total number of data points. First, we embedded the time series ($x\left(t\right)={x}_{1}, {x}_{2}, \dots , {x}_{N}$) of four key spatiotemporal parameters—step length, stride length, step time, and stride time—into an *m*-dimensional state yielding $X\left(t\right)$ [56]. Similar to our previous study [34], we chose the optimal combination of embedding dimension (*m* = 3) and temporal delay (*L* = 1 data point) that was found most sensitive to capture interindividual differences in this cohort. The embedding state-space vectors were thus defined as $X\left(t\right)=\left[{x}_{t},{x}_{t-L},\dots ,{x}_{t-\left(m-1\right)L}\right]=\left[{x}_{t}, {x}_{t-1}{,x}_{t-2}\right]$. Based on the ranks of values within each $X\left(t\right)$, where larger rank corresponds to larger gait parameter value in the examined sequence of three consecutive data points, the distribution of permutation types was determined as follows

3a $$p\left(\pi\right)=\frac{\#\; of\; type\; \pi\; permutation}{\left(T-\left(m-1\right)L+1\right)}$$

Permutation entropy was then calculated according to the definition [57, 58]:

3b $$\begin{array}{cccc}& PE=-\sum\limits_{\pi \epsilon\Pi }p\left(\uppi \right){\mathrm{log}}_{2}p\left(\uppi \right)& & \end{array}$$

where $\Pi$ denotes the set of all possible permutation of ranks in the three-dimensional state space: 1–2-3, 1–3-2, 2-1-3, 2–3-1, 3-1-2, 3-2-1.

PE provides a nonlinear, dynamic perspective on gait control. The notion of entropy of a dynamic system, such as control of gait pattern, relates to information-theoretical, model-free—thus non-linear—measures capturing the temporal complexity of its “behavior”. Specifically, entropy quantifies the *predictability* of time series data, stride sequences in this case. Unlike traditional variability metrics that describe the magnitude of fluctuations, PE evaluates the *temporal orderliness* of those fluctuations over time and captures how sequential gait events evolve dynamically. In the context of gait analysis, PE reflects the richness of temporal patterns within stride sequences, offering insight into the adaptability and flexibility of neuromotor control. Higher PE values indicate more complex and less predictable gait dynamics, typically associated with healthy, adaptable motor control systems capable of responding efficiently to perturbations. Conversely, lower PE values suggest a loss of dynamic richness and increased regularity, which may reflect reduced adaptability of the motor control network, as seen in aging or neurological impairment.

The resulting values provide a robust estimate of gait signal complexity, complementing amplitude-based measures (MAD and CoV) to deliver a multidimensional characterization of gait control. Together, MAD, CoV, and PE provide a comprehensive characterization of gait dynamics, capturing both the *magnitude* and *temporal structure* of stride-to-stride fluctuations. While MAD and CoV quantify the extent of temporal and spatial variability in gait parameters, PE complements these by assessing the underlying complexity and predictability of gait cycles. The combined use of these measures allows for a multidimensional evaluation of locomotor control encompassing stability, consistency, and adaptability, which are all essential features of healthy gait. This integrated approach enables the detection of subtle alterations in gait associated with aging and increased cognitive load, offering sensitive indices of early functional decline that may precede overt mobility impairment. MATLAB 2023a (Mathworks Inc., Natick, MA, USA) was used for subject-level analysis, using custom scripts for calculation of PE.

### Statistical tests

All statistical analyses were performed using MATLAB 2023a. The Lilliefors test was used to assess the normality of data distributions. Since most variables did not follow a normal distribution, results are reported as medians with interquartile ranges (IQR) unless otherwise specified.

To explore associations between chronological age and gait parameters, Spearman’s rank correlation coefficients were computed separately for the single-task and dual-task conditions, as well as for the dual-task cost (DTC). The DTC was defined as the arithmetic difference between a given gait parameter measured under dual-task and single-task conditions, providing an index of cognitive load–related gait alterations.

For group comparisons, participants were categorized into YA and OA. Gait parameters were compared between these groups using the Mann–Whitney *U* test. For parameters exhibiting normal distributions, unpaired two-tailed *t*-tests were applied. Levene’s test was used to assess the homogeneity of variances, and Welch’s correction was applied when variances were unequal.

To evaluate the effects of cognitive load, gait parameters obtained under dual-task conditions were compared with their single-task counterparts using the Wilcoxon signed-rank test. When normality assumptions were met, a paired *t*-test was used instead. All statistical tests were two-tailed, and a significance level of *α* = 0.05 was adopted. To control multiple comparisons when assessing the association of age with the studied gait measure under single and dual task condition, we considered an adjusted threshold for significance defined as *α*_adj_ = 0.05/56 ≈ 0.00089, where 56 is the number of statistical tests of the same type (28 variable × 2 condition). Graphical representations, including boxplots and scatterplots, were generated to visualize group differences, task-related effects, and age-dependent correlations.

## Results

### Participant characteristics

Table 2 summarizes participants’ demographic characteristics, educational attainment, medical history, and medication use, while Table 3 presents baseline physiological parameters and key health indicators. The cohort included 103 adults (45 males, 58 females) aged 23–87 years, divided into younger adults (YA; < 65 years) and older adults (OA; ≥ 65 years) groups. As expected, age groups differed in several baseline physiological and clinical characteristics (Table 2 and 3). As expected, the OA group exhibited higher prevalence of hypertension, dyslipidemia, and polypharmacy. In this pilot analysis, age was treated as the primary independent variable, and statistical comparisons were performed accordingly. Full multivariable adjustment for vascular and metabolic confounders will be implemented in the larger Semmelweis Study cohort.

**Table 2:** Demographic characteristics of the study population

*Characteristics*	**Younger adults (*n* = 62)**		**Older adults (*n* = 41)**	
	***n***	**%**	***n***	**%**
*Sex*				
* Male*	27	26.2	18	17.5
* Female*	35	34.0	23	22.3
*Highest level of education*				
* Academic doctorate degree*	17	16.5	5	4.8
* Professional doctorate degree*	11	10.7	0	0
* Master degree*	20	19.4	11	10.7
* Bachelor degree*	11	10.7	15	14.6
* Other, no college or university degree*	3	2.9	10	9.7
*Handedness*				
* Left*	7	6.8	4	3.9
* Right*	55	53.4	37	35.9

**Table 3:** Health conditions, medication use, and baseline physiological characteristics of the study population

*Diseases, medications and baseline physiological measures*	Younger Adults (***n*** = 62)		Older Adults (***n*** = 41)	
	***n***	%	***n***	%
*Active diseases*				
* Hypertension (controlled)*	5	4.9	23	22.3
* Other cardiovascular diseases*	3	2.9	3	2.9
* Diabetes mellitus (controlled)*	2	1.9	5	4.9
* Hypothyroidism*	1	1.0	2	2.0
* Other metabolic disorder*	2	1.9	6	17.6
* Psychiatric disease*	3	2.9	7	6.8
* Neurological disease*	2	1.9	3	2.9
* Diseases of bones, joints and muscle*	7	3.9	13	12.5
* Other medical conditions*	7	6.8	12	11.7
*Current smoker*	6	5.8	6	5.8
*Medications*				
* AT1-receptor blocker*	1	1.0	6	5.8
* ACE-inhibitor*	1	1.0	7	6.8
* β-blocker*	1	1.0	7	6.8
* Ca-antagonist*	2	1.9	7	6.8
* Diuretic*	3	2.9	10	9.7
* Statin*	2	1.9	12	11.7
* Estrogen supplement*	1	1.0	6	3.9
* Thyroid supplement*	1	1.0	11	10.7
* Other prescribed drugs*	22	21.4	26	25.2
*Baseline data*	Mean	SD	Mean	SD
* Systolic blood pressure*	116.4	12.8	126.3	16.2
* Diastolic blood pressure*	77.6	10.3	74.9	8.5
* Mean arterial blood pressure*	90.5	10.7	92.1	8.6
* Heart rate*	66.0	9.8	64.1	13.1
* Body mass index (BMI)*	25.3	5.3	26.6	4.3

### MAD of gait parameters

Analysis of the MAD—quantified for the right leg—revealed robust age- and task-related effects across multiple gait domains. Table 4 shows age-stratified gait variability and complexity metrics of key spatiotemporal gait parameters.

**Table 4:** Age-stratified gait variability and complexity metrics, including the median absolute deviation (MAD), coefficient of variation (CoV), and permutation entropy (PE) of key spatiotemporal gait parameters measured under single- and dual-task conditions. Values represent the median [interquartile range] and basic descriptive statistics for each gait parameter. Abbreviations: YA = younger adults (< 65 years); OA = older adults (≥ 65 years); *MAD*, median absolute deviation; *CoV*, coefficient of variation; *PE*, permutation entropy; *CoP*, center of pressure

Gait parameter Median [IQR]	Median absolute deviation				Coefficient of variation				Permutation entropy			
	**Single task**		**Dual task**		**Single task**		**Dual task**		**Single task**		**Dual task**	
	**YA**	**OA**	**YA**	**OA**	**YA**	**OA**	**YA**	**OA**	**YA**	**OA**	**YA**	**OA**
*Step time (s)*	0.009 [0.008]	0.009 [0.007]	0.0160 [0.008]	0.017 [0.013]	0.095 [0.059]	0.098 [0.059]	0.010 [0.059]	0.114 [0.073]	0.064 [0.021]	0.085 [0.052]	0.070 [0.032]	0.108 [0.092]
*Stride Time (s)*	0.017 [0.010]	0.017 [0.008]	0.025 [0.013]	0.027 [0.021]	0.452 [0.789]	0.445 [0.800]	0.563 [0.888]	0.409 [1.195]	0.619 [0.108]	0.651 [0.086]	0.623 [0.091]	0.647 [0.084]
*Step Length (cm)*	1.361 [0.575]	1.577 [0.641]	1.477 [0.619]	1.752 [0.762]	0.037 [0.016]	0.050 [0.013]	0.040 [0.008]	0.059 [0.027]	0.555 [0.168]	0.635 [0.119]	0.588 [0.114]	0.652 [0.129]
*Stride Length (cm)*	2.199 [0.981]	2.497 [1.149]	2.377 [1.143]	3.167 [1.647]	0.030 [0.011]	0.039 [0.015]	0.033 [0.012]	0.052 [0.034]	0.636 [0.089]	0.688 [0.088]	0.625 [0.094]	0.672 [0.098]
*Stride Width (m)*	1.395 [0.431]	1.694 [0.504]	1.466 [0.540]	1.739 [0.842]	0.292 [0.179]	0.354 [0.389]	0.269 [0.186]	0.367 [0.370]				
*Stance (%)*	0.641 [0.242]	0.712 [0.272]	0.716 [0.256]	0.843 [0.402]	0.035 [0.011]	0.038 [0.013]	0.037 [0.011]	0.051 [0.035]				
*Single Support (%)*	0.603 [0.189]	0.766 [0.228]	0.685 [0.254]	0.881 [0.546]	0.030 [0.018]	0.033 [0.015]	0.032 [0.022]	0.056 [0.064]				
*Total Double Support (%)*	0.779 [0.289]	0.924 [0.276]	0.805 [0.311]	0.992 [0.513]	0.017 [0.008]	0.021 [0.008]	0.019 [0.008]	0.029 [0.038]				
*Integrated Pressure (Pa∙s)*	6.317 [3.072]	7.213 [4.109]	7.363 [2.798]	9.179 [5.203]	0.030 [0.016]	0.040 [0.024]	0.034 [0.016]	0.063 [0.084]				
*Stance CoP distance (%)*	0.847 [0.696]	0.979 [0.723]	0.845 [0.684]	1.164 [0.721]	0.029 [0.004]	0.037 [0.012]	0.031 [0.001]	0.053 [0.032]				
*SingleSupport CoP distance*	1.470 [0.429]	1.614 [0.667]	1.581 [0.623]	1.925 [0.957]	0.044 [0.011]	0.047 [0.008]	0.047 [0.014]	0.053 [0.039]				

#### Temporal parameters

**Fig. 1:**
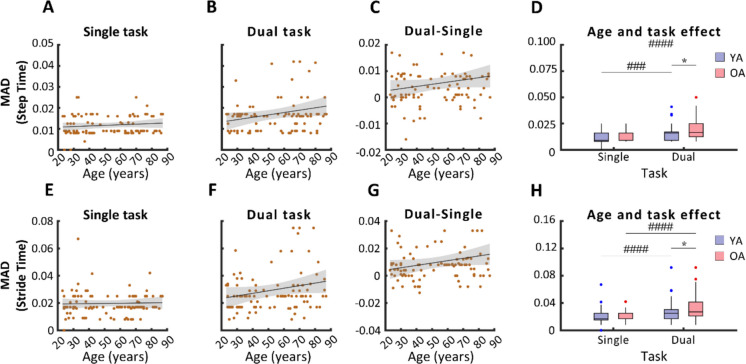
Relationship between age and the median absolute deviation (MAD) of temporal gait parameters during single- and dual-task walking. Panels **A**–**D** show results for step time, and panels **E**–**H** for stride time. Black trend lines with gray shaded areas indicate the fitted regression and its 95% confidence interval. Box-and-whisker plots display data for younger (*n* = 60) and older (*n* = 41) participants, shown in light blue and light red, respectively. Blue and red dots mark statistical outliers. **A** Age-related changes in the MAD for step time during single-task walking. **B** The MAD for step time during dual-task walking significantly increases with age (Spearman’s *p* < 0.05). **C** Difference in MAD values between single- and dual-task conditions as a function of age. **D** Combined effects of age group and task condition on the MAD for step time (*p < 0.05, age effect; ####*p* < 0.0001, task effect). **E** Age-related changes in MAD for stride time during single-task walking. **F** The MAD for stride time during dual-task walking significantly increases with age (Spearman’s *p* < 0.05). **G** Difference in MAD values between single- and dual-task conditions as a function of age. **H** Combined effects of age group and task condition on the MAD for stride time (*p < 0.05, age effect; ###*p* < 0.0001, ####*p* < 0.0001, task effect; Mann–Whitney and Wilcoxon tests)

Generally, MAD of step time and stride time increased with age; however, these relationships were not significant for single task data. During dual tasks, MAD of stride time exhibited significant positive correlation with age (Fig. 1F, *ρ* = 0.197, *p* = 0.0484). In line with this finding, MAD corresponding to dual task condition was significantly higher in OA compared to YA for step time (Fig. 1D, p = 0.033) and for stride time (Fig. 1H, p = 0.012); however, the two age groups were not different during single task for any of these parameters. The effect of dual task on these temporal variability parameters was significant (Fig. 1D**, **Fig. 1H), for all MAD values (*p* < 0.0001 for both age groups and for both parameters, expect for step time in YA with *p* = 0.0002).

#### Spatial parameters

**Fig. 2:**
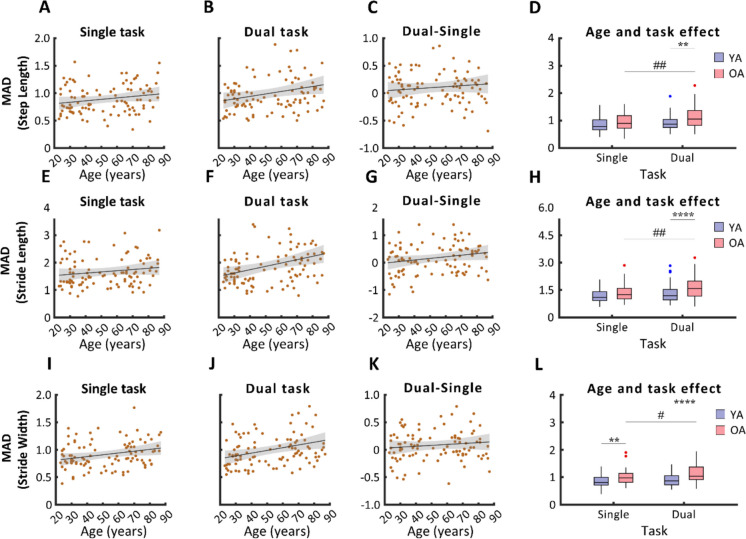
Relationship between age and the median absolute deviation (MAD) of height-normalized spatial gait parameters during single- and dual-task walking. Panels **A**–**D** show results for step length, **E**–**H** for stride length, and **I**–**L** for stride width. Black trend lines with gray shaded areas indicate the fitted regression and its 95% confidence interval. Box-and-whisker plots display data for younger (*n* = 60) and older (*n* = 41) participants, shown in light blue and light red, respectively. Blue and red dots mark statistical outliers. **A** Age-related changes in the median absolute deviation for step length during single-task walking. **B** The median absolute deviation for step length during dual-task walking significantly increases with age (Spearman’s *p* < 0.05). **C** Difference in median absolute deviation between single- and dual-task conditions as a function of age. **D** Combined effects of age group and task condition on the median absolute deviation for step length (**p < 0.01, age effect; ##*p* < 0.01, task effect; Mann–Whitney and Wilcoxon tests). **E** Age-related changes in the median absolute deviation for stride length during single-task walking. **F** The median absolute deviation for stride length during dual-task walking significantly increases with age (Spearman’s *p* < 0.05). **G** Difference in median absolute deviation between single- and dual-task conditions as a function of age. **H** Combined effects of age group and task condition on the median absolute deviation for stride length (****p < 0.0001, age effect; ##*p* < 0.01, task effect; Mann–Whitney and Wilcoxon tests). **I** Age-related changes in the median absolute deviation for stride width during single-task walking (Spearman’s *p* < 0.05). **J** The median absolute deviation for stride width during dual-task walking significantly increases with age (Spearman’s *p* < 0.05). **K** Difference in median absolute deviation between single- and dual-task conditions as a function of age. **L** Combined effects of age group and task condition on the median absolute deviation for stride width (**p < 0.01, ****p < 0.0001, age effect; #*p* < 0.05, task effect; Mann–Whitney and Wilcoxon tests)

The relationship between age and MAD of height-normalized stride width was the only significant association (Fig. 2I, *ρ* = 0.244, *p* = 0.014) for single task condition data. MAD of height-normalized step length during dual task condition positively correlated with age (Fig. 2B, *ρ* = 0.230, *p* = 0.021). Based on spatial gait parameters, the two age groups significantly differed only in case of height-normalized stride width variability (Fig. 2L, p = 0.002). The effect of age group is significant for dual task in case of all spatial MAD measures (Fig. 2D, step length: *p* = 0.005; Fig. 2H and L, stride length and stride width: *p* < 0.0001, respectively). All spatial parameters exhibited significantly greater median MAD values in the dual task condition compared to single task condition.

#### Gait cycle parameters

**Fig. 3:**
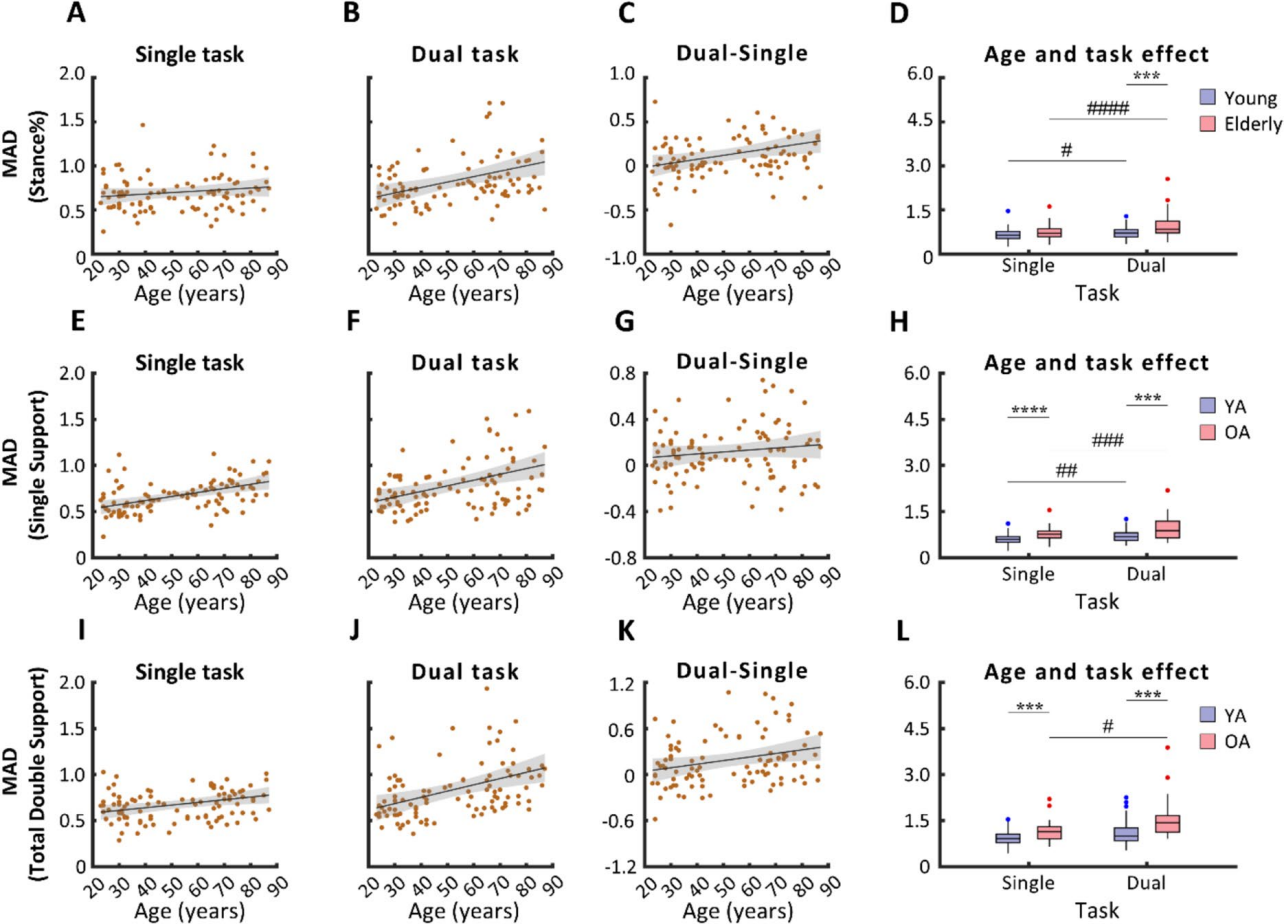
Relationship between age and the median absolute deviation (MAD) of gait cycle parameters during single- and dual-task walking. Panels **A**–**D** show results for stance phase, **E**–**H** for single support, and **I**–**L** for total double support. Black trend lines with gray shaded areas indicate the fitted regression and its 95% confidence interval. Box-and-whisker plots display data for younger (*n* = 60) and older (*n* = 41) participants, shown in light blue and light red, respectively. Blue and red dots mark statistical outliers. **A** Age-related changes in MAD for stance phase during single-task walking. **B** MAD for stance phase during dual-task walking significantly increases with age (Spearman’s *p* < 0.05). **C** Difference in MAD between single- and dual-task conditions as a function of age. **D** Combined effects of age group and task condition on MAD for stance phase (***p < 0.001, age effect; #*p* < 0.05, ####*p* < 0.0001, task effect; Mann–Whitney and Wilcoxon tests). **E** Age-related changes in MAD for single support during single-task walking (Spearman’s *p* < 0.05). **F** MAD for single support during dual-task walking significantly increases with age (Spearman’s *p* < 0.05). **G** Difference in MAD between single- and dual-task conditions as a function of age. **H** Combined effects of age group and task condition on MAD for single support (****p < 0.0001, age effect; ##*p* < 0.01, task effect; Mann–Whitney and Wilcoxon tests). **I** Age-related changes in MAD for total double support during single-task walking (Spearman’s *p* < 0.05). **J** MAD for total double support during dual-task walking significantly increases with age (Spearman’s *p* < 0.05). (K) Difference in MAD between single- and dual-task conditions as a function of age. **L** Combined effects of age group and task condition on MAD for total double support (***p < 0.001, age effect; #*p* < 0.05, task effect; Mann–Whitney and Wilcoxon tests)

Variability of gait cycle parameters demonstrated strong age-related changes (Fig. 3). Stance phase duration increased significantly with age during dual task conditions (Fig. 3B, *ρ* = 0.357, *p* < 0.0001), which relationship was significant for the differences with single task MAD values. Association with age was significant during single task condition for single support (Fig. 3E, *ρ* = 0.482, *p* < 0.0001) and total double support (Fig. 3I, *ρ* = 0.319, *p* < 0.0001). MAD of these parameters during dual task also positively correlated with age as shown in Fig. 3F (single support, *ρ* = 0.358, *p* < 0.0001) and in Fig. 3J (total double support, *ρ* = 0.266, *p* = 0.007).

Comparisons between age groups revealed significant differences in MAD of stance during dual talk (Fig. 3D, p = 0.0005) and in MAD of all other gait cycle parameters for both task conditions (Figs. 3H and 3L, p < 0.001 for all). Performing mental arithmetics increased MAD of all of these parameters indicating significant task effect in both age groups for stance (Fig. 3D, YA: *p* = 0.028; OA: *p* < 0.0001), for single support (Fig. 3H, YA: *p* = 0.0014; OA: *p* = 0.0009) and in OA for total double support (Fig. 3L, p = 0.0223).

#### Balance control parameters

**Fig. 4:**
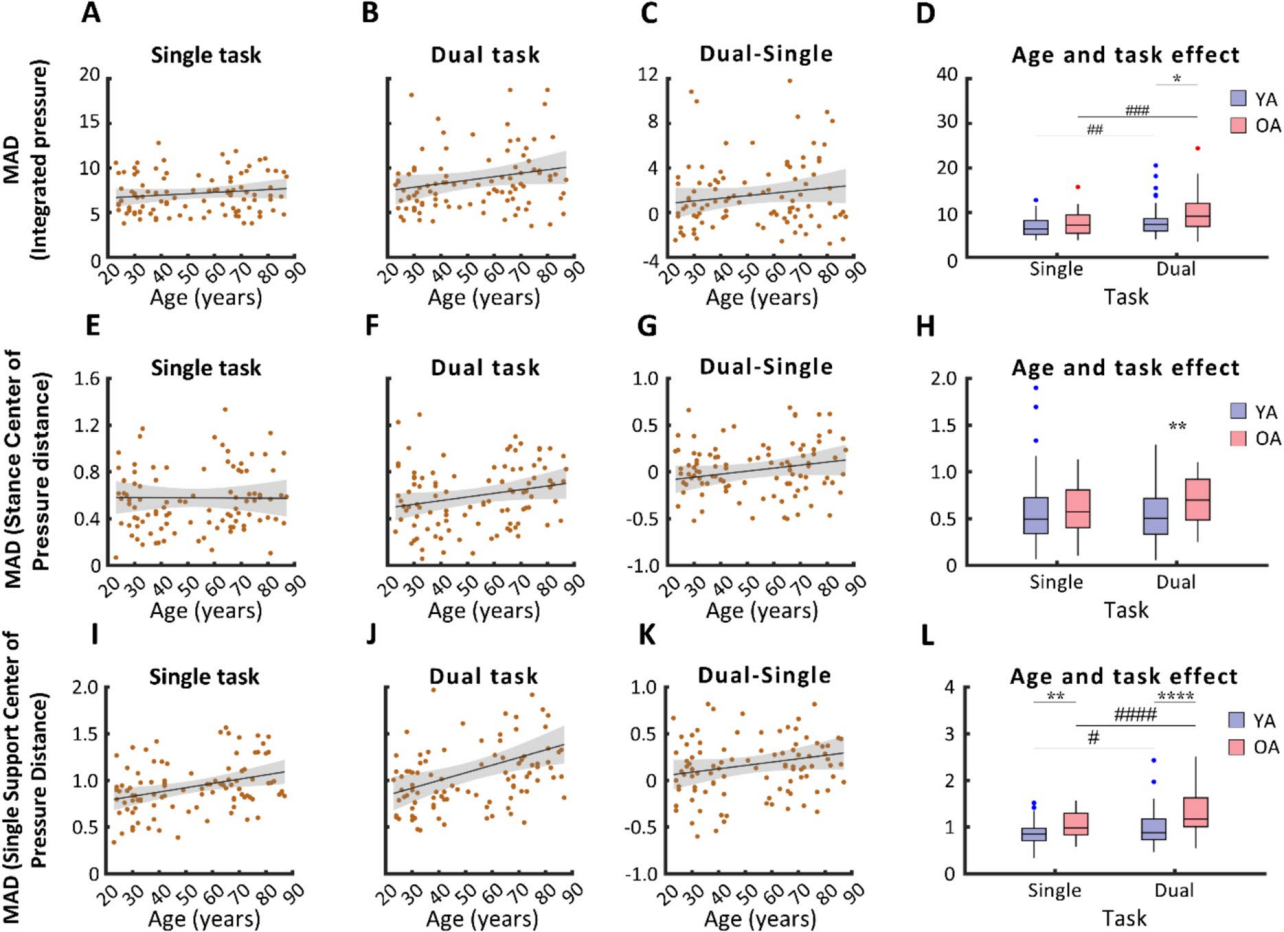
Relationship between age and the median absolute deviation (MAD) of gait parameters reflecting balance control during single- and dual-task walking. Panels **A**–**D** show results for integrated pressure, (E–H) for stance center of pressure distance, and **I**–**L** for single support center of pressure distance. Black trend lines with gray shaded areas indicate the fitted regression and its 95% confidence interval. Box-and-whisker plots display data for younger (*n* = 60) and older (*n* = 41) participants, shown in light blue and light red, respectively. Blue and red dots mark statistical outliers. **A** Age-related changes in MAD for integrated pressure during single-task walking. **B** MAD for integrated pressure during dual-task walking significantly increases with age (Spearman’s *p* < 0.05). **C** Difference in MAD between single- and dual-task conditions as a function of age. **D** Combined effects of age group and task condition on MAD for integrated pressure (*p < 0.05, age effect; ##*p* < 0.01, ##*p* < 0.001, task effect; Mann–Whitney and Wilcoxon tests). **E** Age-related changes in MAD for stance center of pressure distance during single-task walking. **F** MAD for stance center of pressure distance during dual-task walking significantly increases with age (Spearman’s *p* < 0.05). **G** Difference in MAD between single- and dual-task conditions as a function of age. **H** Combined effects of age group and task condition on MAD for stance center of pressure distance (**p < 0.01, age effect; Mann–Whitney test). **I** Age-related changes in MAD for single support center of pressure distance during single-task walking (Spearman’s *p* < 0.05). **J** MAD for single support center of pressure distance during dual-task walking significantly increases with age (Spearman’s *p* < 0.05). **K** Difference in MAD between single- and dual-task conditions as a function of age. **L** Combined effects of age group and task condition on MAD for single support center of pressure distance (**p < 0.01, ****p < 0.0001, age effect; #*p* < 0.05, ####*p* < 0.0001, task effect; Mann–Whitney and Wilcoxon tests)

Figure 4 illustrates the relationship between age and MAD of the following gait parameters: integrated pressure, stance center of pressure (CoP) distance and single support CoP distance under single- and dual-task conditions. Variability of integrated pressure significantly increased with age only during dual task conditions (Fig. 4B) corresponding to significant difference between OA and YA (Fig. 4D, p = 0.0103). The difference between walking conditions was significant (Fig. 4D, YA: *p* = 0.0047; OA: *p* = 0.0009).

Similarly, the relationship between age or age group and MAD of stance CoP distance was only significant for dual task (Fig. 4E, *ρ* = 0.231, *p* = 0.0199; Fig. 4H, p = 0.0029). However, stance CoP distance variability did not significantly differ between single and dual task condition in neither age groups.

MAD of single support significantly increased with age in both task conditions (Fig. 4I, single: *ρ* = 0.32, *p* = 0.0011; Fig. 4J, dual: *ρ* = 0.401, *p* < 0.0001). Comparing age groups, variability of single support CoP distance was significantly lower in OA compared to YA (Fig. 4L) during single- (*p* = 0.0012) and dual-task conditions (*p* = 0.0001). Furthermore, dual task conditions significantly reduced single support CoP distance in YA (*p* = 0.0316) and in OA (*p* < 0.0001), further highlighting the impact of cognitive load on balance control.

### CoV of gait parameters

CoV values revealed similar patterns than MAD, confirming that gait variability increased with both aging and cognitive load; however, certain group effects and associations were notably different.

#### Temporal parameters

**Fig. 5:**
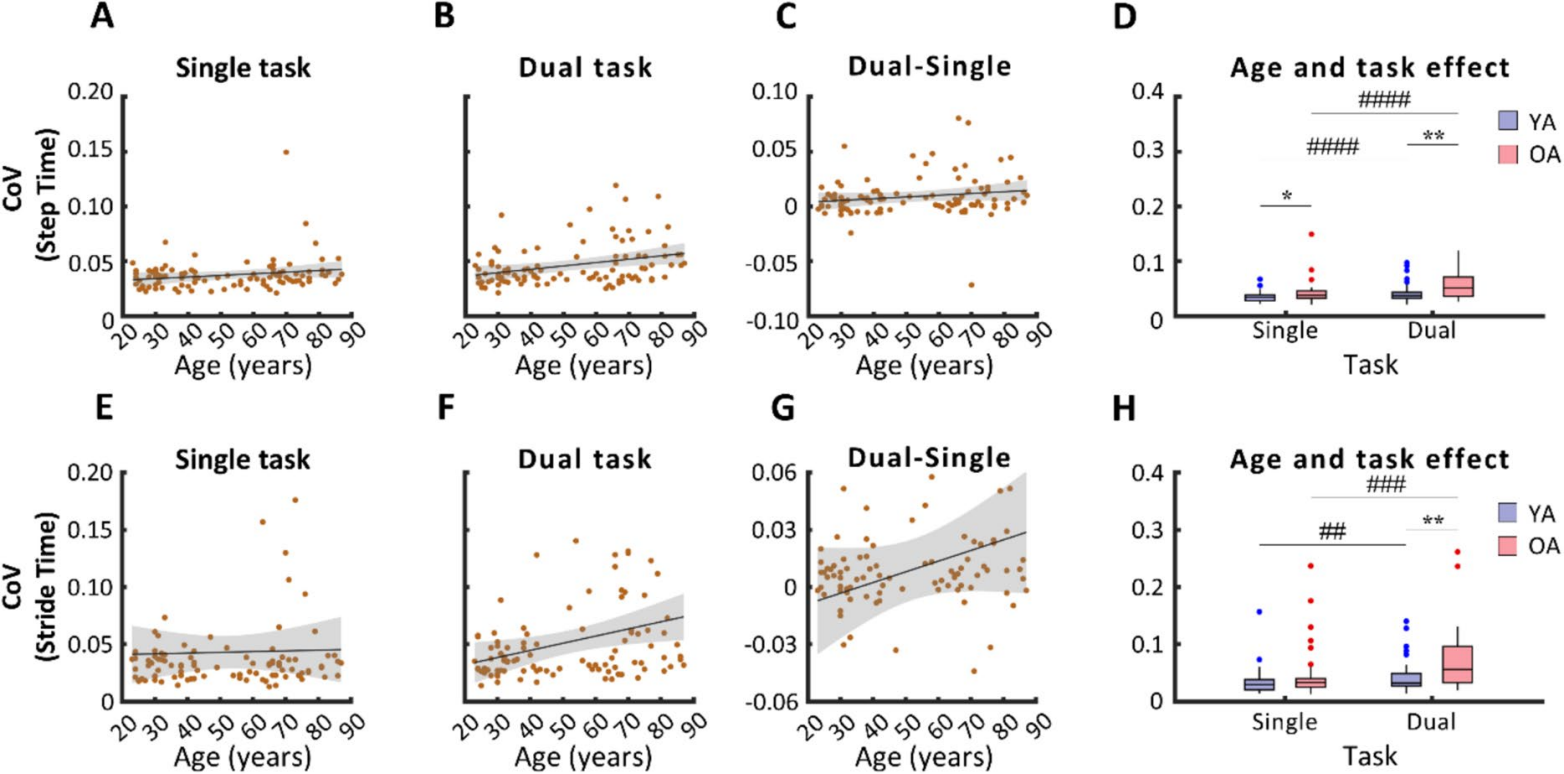
Relationship between age and the coefficient of variation (CoV) of temporal gait parameters during single- and dual-task walking. Panels **A**–**D** show results for step time, and panels **E**–**H** for stride time. Black trend lines with gray shaded areas indicate the fitted regression and its 95% confidence interval. Box-and-whisker plots display data for younger (*n* = 60) and older (*n* = 41) participants, shown in light blue and light red, respectively. Blue and red dots mark statistical outliers. **A** CoV for step time during single-task walking significantly increases with age (Spearman’s *p* < 0.05). **B** CoV for step time during dual-task walking significantly increases with age (Spearman’s *p* < 0.05). **C** Difference in CoV between single- and dual-task conditions as a function of age. **D** Combined effects of age group and task condition on CoV for step time (*p < 0.05, **p < 0.01, age effect; ####*p* < 0.0001, task effect; Mann–Whitney and Wilcoxon tests). **E** Age-related changes in CoV for stride time during single-task walking. **F** CoV for stride time during dual-task walking significantly increases with age (Spearman’s *p* < 0.05). **G** Difference in CoV between single- and dual-task conditions as a function of age. **H** Combined effects of age group and task condition on CoV for stride time (**p < 0.01, age effect; ##*p* < 0.01, ###*p* < 0.001, task effect; Mann–Whitney and Wilcoxon tests)

The CoV of step time exhibited a significant age-related increase under dual-task conditions (Fig. 5B, *ρ* = 0.319, *p* = 0.001) and was significantly higher among older adults (Fig. 5D) during single (*p* = 0.012) and dual task condition (*p* = 0.001). Similar trends were observed for the CoV of stride time (Fig. 5F, *ρ* = 0.298, *p* = 0.001), but, in addition, dual-task cost also correlated with chronological age (*ρ* = 0.199, *p* = 0.049). Task effect is significant for step time (Fig. 5D, p < 0.0001 for both age groups) and stride time (Fig. 5H, p = 0.005 for YA and *p* < 0.0001 for OA).

#### Spatial parameters

**Fig. 6:**
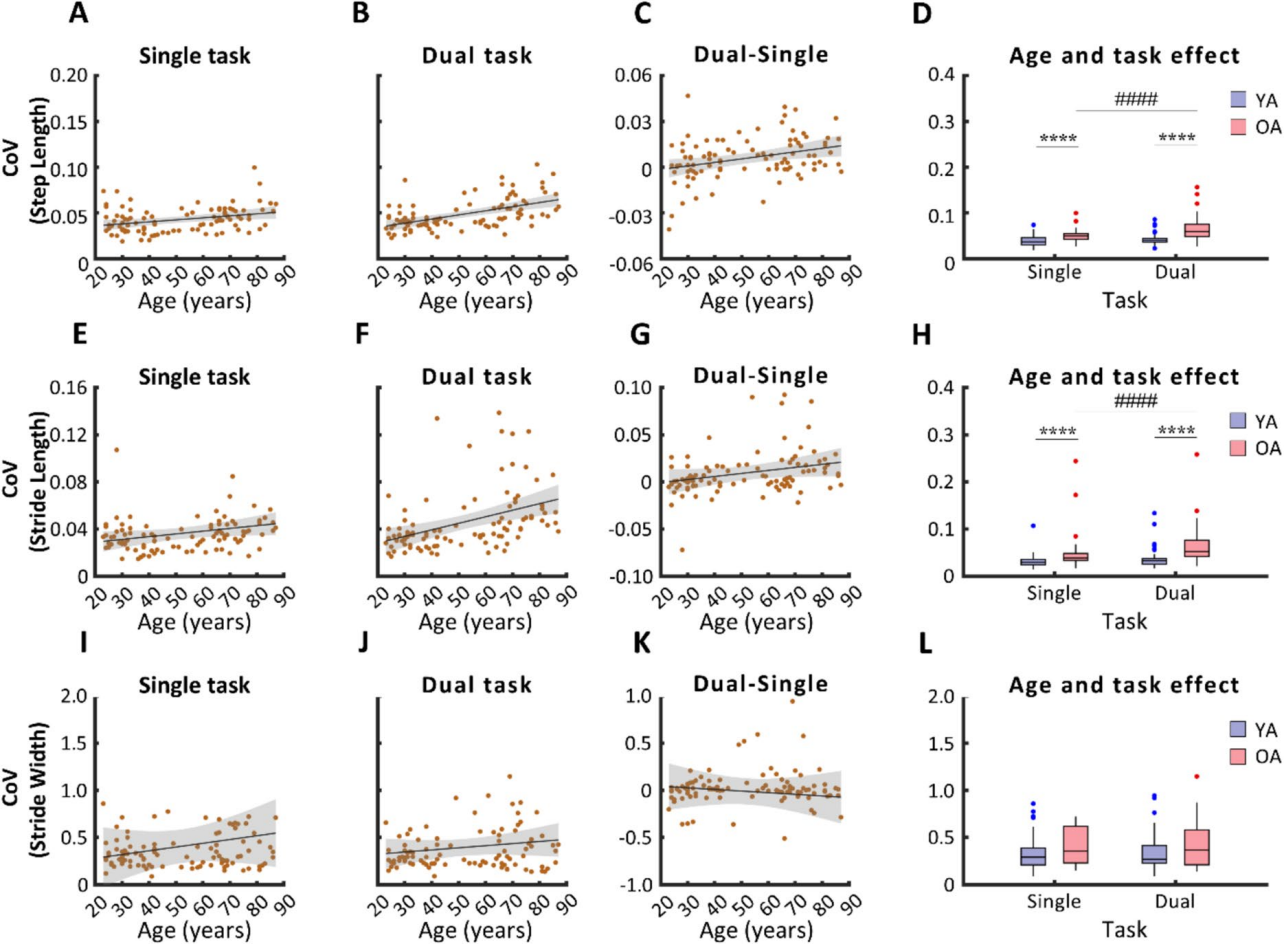
Relationship between age and the coefficient of variation (CoV) of spatial gait parameters during single- and dual-task walking. Panels **A**–**D** show results for step length, **E**–**H** for stride length, and **I**–**L** for stride width. Black trend lines with gray shaded areas indicate the fitted regression and its 95% confidence interval. Box-and-whisker plots display data for younger (*n* = 60) and older (*n* = 41) participants, shown in light blue and light red, respectively. Blue and red dots mark statistical outliers. **A** CoV for step length during single-task walking significantly increases with age (Spearman’s *p* < 0.05). **B** CoV for step length during dual-task walking significantly increases with age (Spearman’s *p* < 0.05). **C** Difference in CoV between single- and dual-task conditions as a function of age (Spearman’s *p* < 0.05). **D** Combined effects of age group and task condition on CoV for step length (****p < 0.0001, age effect; ####*p* < 0.0001, task effect; Mann–Whitney and Wilcoxon tests). **E** CoV for stride length during single-task walking significantly increases with age (Spearman’s *p* < 0.05). **F** CoV for stride length during dual-task walking significantly increases with age (Spearman’s *p* < 0.05). **G** Difference in CoV between single- and dual-task conditions as a function of age (Spearman’s *p* < 0.05). **H** Combined effects of age group and task condition on CoV for stride length (****p < 0.0001, age effect; ####*p* < 0.0001, task effect; Mann–Whitney and Wilcoxon tests). **I** Age-related changes in CoV for stride width during single-task walking. **J** Age-related changes in CoV for stride width during dual-task walking. **K** Difference in CoV between single- and dual-task conditions as a function of age. **L** Combined effects of age group and task condition on CoV for stride width

The CoV of step length markedly increased with age in case of single (Fig. 6A, *ρ* = 0.323, *p* = 0.001) and dual task (Fig. 6B, *ρ* = 0.554, *p* < 0.0001) conditions and the difference between age group were significant accordingly (Fig. 6D, p < 0.0001 for both task conditions). Dual task significantly increased the CoV of step length compared to single task only in the OA group (Fig. 6D, p < 0.0001); however, this difference showed a significant association with age (Fig. 6C, *ρ* = 0.363, *p* = 0.0002). We found a statistically similar association between age and CoV of stride length during single (Fig. 6E, *ρ* = 0.318, *p* = 0.001) and dual task (Fig. 6F, *ρ* = 0.549, *p* < 0.0001), as well as for the difference between these walking conditions (Fig. 6G, *ρ* = 0.338, *p* = 0.0005). This similarity to CoV of step length is further illustrated by Fig. 6H demonstrating a pronounced significant difference between age groups in both task conditions (*p* < 0.0001) and a strong effect of task on this gait variability parameter (*p* < 0.0001) in the OA group. In striking contrast, CoV of stride width does not associate with age, and does no differ across age groups and walking conditions (Figs. 6I-6–L).

#### Gait cycle parameters

**Fig. 7:**
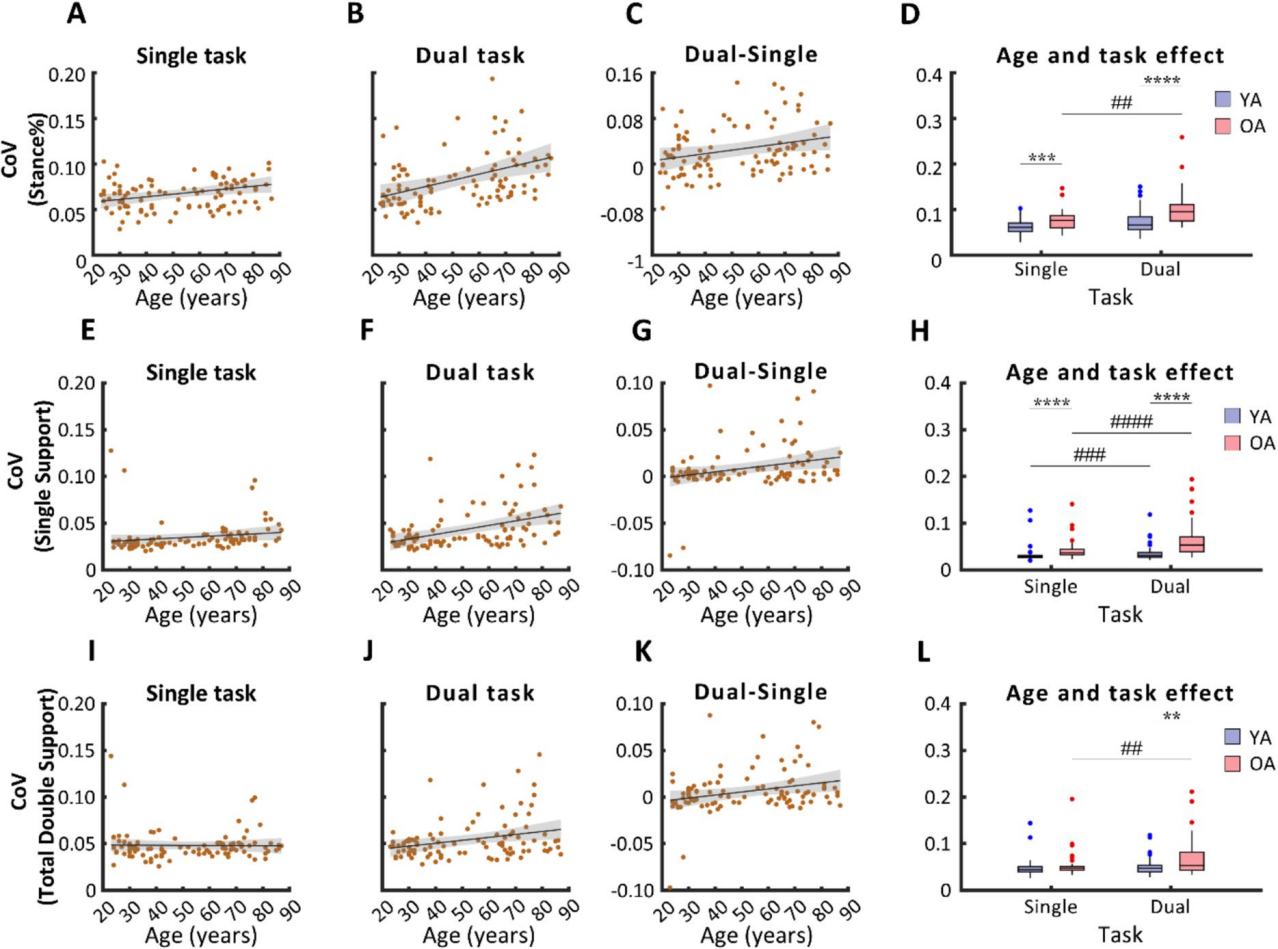
Relationship between age and the coefficient of variation (CoV) of gait cycle parameters during single- and dual-task walking. Panels **A**–**D** show results for stance phase, **E**–**H** for single support, and **I**–**L** for total double support. Black trend lines with gray shaded areas indicate the fitted regression and its 95% confidence interval. Box-and-whisker plots display data for younger (*n* = 60) and older (*n* = 41) participants, shown in light blue and light red, respectively. Blue and red dots mark statistical outliers. (A) CoV for stance phase during single-task walking significantly increases with age (Spearman’s *p* < 0.05). **B** CoV for stance phase during dual-task walking significantly increases with age (Spearman’s *p* < 0.05). **C** Difference in CoV between single- and dual-task conditions as a function of age (Spearman’s *p* < 0.05). **D** Combined effects of age group and task condition on CoV for stance phase (**p < 0.01, ***p < 0.001, age effect; ##*p* < 0.01, task effect; Mann–Whitney and Wilcoxon tests). **E** CoV for single support during single-task walking significantly increases with age (Spearman’s *p* < 0.05). **F** CoV for single support during dual-task walking significantly increases with age (Spearman’s *p* < 0.05). **G** Difference in CoV between single- and dual-task conditions as a function of age (Spearman’s *p* < 0.05). **H** Combined effects of age group and task condition on CoV for single support (***p < 0.001, age effect; ###*p* < 0.001, ####*p* < 0.0001, task effect; Mann–Whitney and Wilcoxon tests). **I** Age-related changes in CoV for total double support during single-task walking. **J** CoV for total double support during dual-task walking significantly increases with age (Spearman’s *p* < 0.05). **K** Difference in CoV between single- and dual-task conditions as a function of age (Spearman’s *p* < 0.05). **L** Combined effects of age group and task condition on CoV for total double support (**p < 0.01, age effect; ##*p* < 0.01, task effect; Wilcoxon test)

CoV of gait cycle parameters demonstrated a strong association with age. In case of single task, we found significant correlations for stance (Fig. 7A, *ρ* = 0.221, *p* = 0.028) and single support (Fig. 7E, *ρ* = 0.489, *p* < 0.0001), but not for total double support. Analysis of gait variability during dual task revealed a stronger correlation for stance (Fig. 7B, *ρ* = 0.221, *p* = 0.028) and single support (Fig. 7F, *ρ* = 0.489, *p* < 0.0001), and for total double support (Fig. 7J, *ρ* = 0.235, *p* = 0.019). Correlating the dual task-induced variability increase with age revealed a significant relationship for stance (Fig. 7C, *ρ* = 0.241, *p* = 0.0164), for single support (Fig. 7G, *ρ* = 0.284, *p* = 0.0042) and for total double support (Fig. 7K, *ρ* = 0.247, *p* = 0.0131).

We found that OA had significantly greater CoV of stance (Fig. 7D) than YA for the single and dual task condition (*p* = 0.0003 and *p* < 0.0001, respectively), while task effect was significant only in OA (*p* = 0.0001). In case of CoV of single support (Fig. 7H), age groups showed a pronounced significant difference (*p* < 0.0001 for both conditions) and task had a significant effect in YA (*p* = 0.001) and OA (*p* < 0.0001). Finally, CoV of total double support (Fig. 7L) during dual task was significantly higher in OA (*p* = 0.009) compared to YA, and during dual task compared to single task only in the OA group (*p* = 0.007).

#### Balance control parameters

**Fig. 8:**
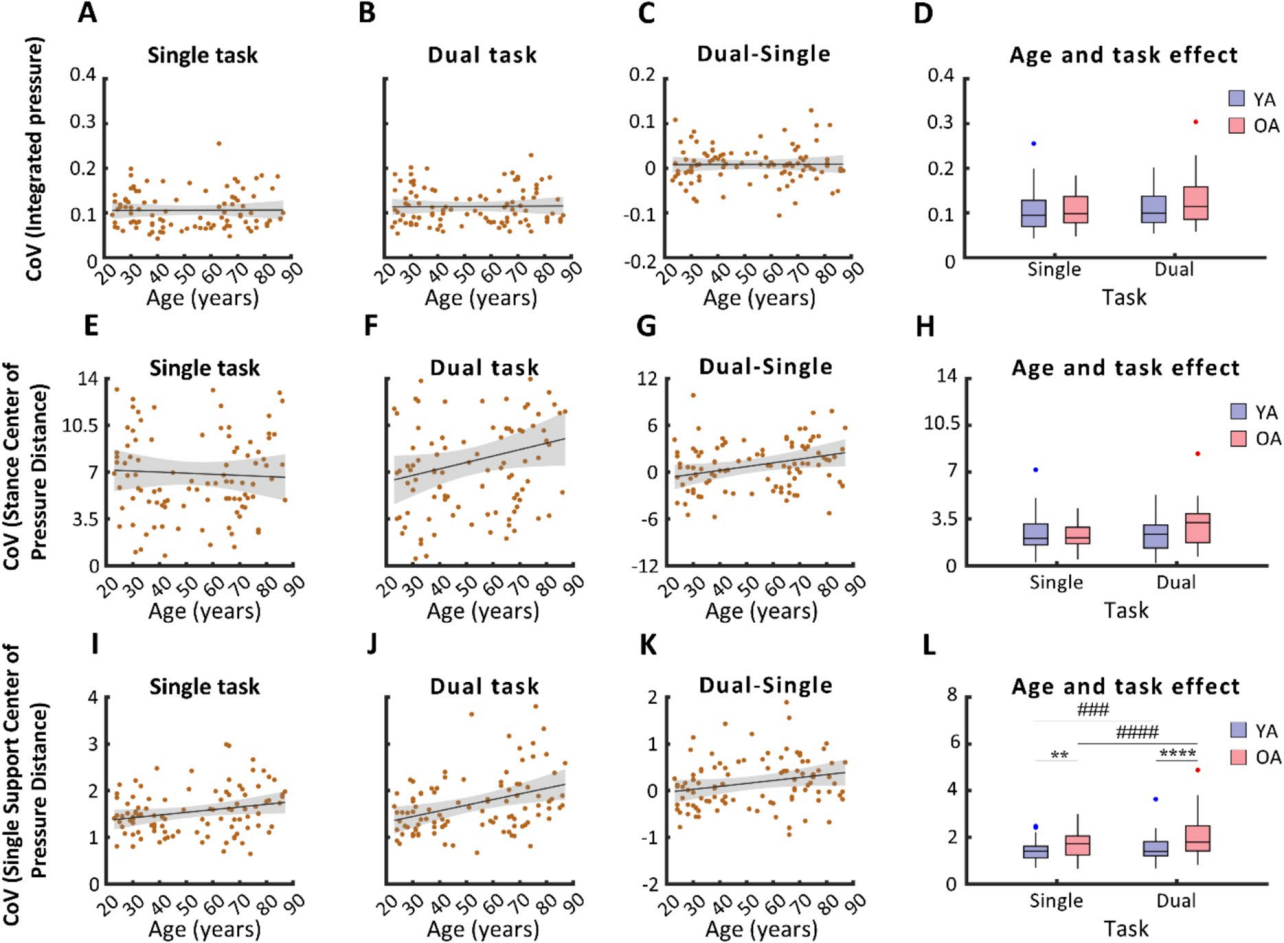
Relationship between age and the coefficient of variation (CoV) of parameters reflecting balance control during single- and dual-task walking. Panels **A**–**D** show results for integrated pressure, **E**–**H** for stance center of pressure distance, and **I**–**L** for single support center of pressure distance. Black trend lines with gray shaded areas indicate the fitted regression and its 95% confidence interval. Box-and-whisker plots display data for younger (*n* = 60) and older (*n* = 41) participants, shown in light blue and light red, respectively. Blue and red dots mark statistical outliers. **A** Age-related changes in CoV for integrated pressure during single-task walking. **B** Age-related changes in CoV for integrated pressure during dual-task walking. **C** Difference in CoV between single- and dual-task conditions as a function of age. **D** Combined effects of age group and task condition on CoV for integrated pressure. **E** Age-related changes in CoV for stance center of pressure distance during single-task walking. **F** Age-related changes in CoV for stance center of pressure distance during dual-task walking. **G** Difference in CoV between single- and dual-task conditions as a function of age. **H** Combined effects of age group and task condition on CoV for stance center of pressure distance. **I** CoV for single support center of pressure distance during single-task walking significantly increases with age (Spearman’s *p* < 0.05). **J** CoV for single support center of pressure distance during dual-task walking significantly increases with age (Spearman’s *p* < 0.05). **K** Difference in CoV between single- and dual-task conditions as a function of age (Spearman’s *p* < 0.05). **L** Combined effects of age group and task condition on CoV for single support center of pressure distance (**p < 0.01, ****p < 0.0001, age effect; ###*p* < 0.001, ####*p* < 0.0001, task effect; Mann–Whitney and Wilcoxon tests)

Among these parameters, only CoV of single support CoP distance correlated significantly with age (single task: Fig. 8I, *ρ* = 0.374, *p* < 0.0001; dual task: Fig. 8J, *ρ* = 0.48, *p* < 0.0001). Figure 8K shows that the difference between dual task and markedly increased with age (*ρ* = 0.408, *p* < 0.0001). Group comparisons (Fig. 8L) revealed a significant effect of age during single and dual task (*p* = 0.0015 and *p* < 0.0001, respectively) only for single support CoP distance CoV values, which were also significantly impacted by task for both age groups (*p* < 0.0001). Integrated pressure CoV values and stance center of pressure CoV values did not significantly correlate with age in any task conditions.

### Permutation entropy of spatiotemporal gait pattern

The PE analysis revealed that gait complexity also increased with age and cognitive load, particularly in temporal parameters.

#### Temporal parameters

**Fig. 9:**
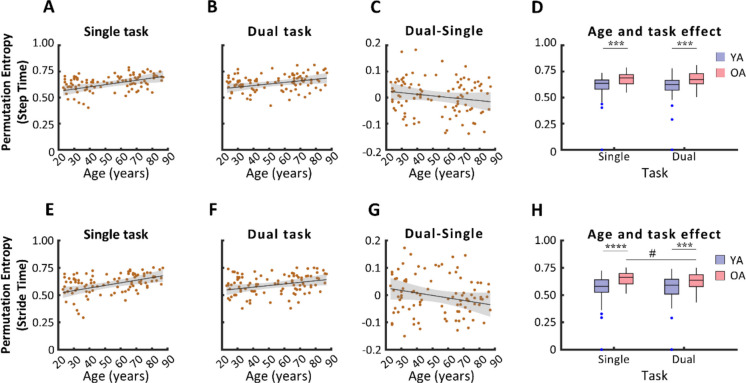
Relationship between age and permutation entropy (PE) of temporal gait parameters during single- and dual-task walking. Panels **A**–**D** show results for step time, and panels **E**–**H** for stride time. Black trend lines with gray shaded areas indicate the fitted regression and its 95% confidence interval. Box-and-whisker plots display data for younger (*n* = 60) and older (*n* = 41) participants, shown in light blue and light red, respectively. Blue and red dots mark statistical outliers. **A** PE for step time during single-task walking significantly increases with age (Spearman’s *p* < 0.05). **B** PE for step time during dual-task walking significantly increases with age (Spearman’s *p* < 0.05). **C** Difference in PE between single- and dual-task conditions as a function of age. **D** Combined effects of age group and task condition on PE for step time (***p < 0.001, age effect; Mann–Whitney test). **E** PE for stride time during single-task walking significantly increases with age (Spearman’s *p* < 0.05). **F** PE for stride time during dual-task walking significantly increases with age (Spearman’s *p* < 0.05). **G** Difference in PE between single- and dual-task conditions as a function of age. **H** Combined effects of age group and task condition on PE for stride time (***p < 0.001, ****p < 0.0001, age effect; #*p* < 0.05, task effect; Mann–Whitney and Wilcoxon tests)

Step time PE values rose significantly with age in both single- (Fig. 9A, *ρ* = 0.4, *p* < 0.0001) and dual-task conditions (Fig. 9B, *ρ* = 0.276, *p* = 0.0063). Correspondingly, we found significantly higher PE values in OA for both single and dual task (*p* = 0.0002 and *p* = 0.0006, respectively), but PE was not different between walking conditions. Figure 9E and F demonstrate similar age-related increase of PE of stride length (single task: *ρ* = 0.380, *p* = 0.0001; dual task: *ρ* = 0.221, *p* = 0.0317) which is also reflected by the significant difference between OA and YA (Fig. 9H: single task: *p* < 0.0001; dual task: *p* = 0.0001). Stride length PE was significantly higher during dual task compared to single task condition (Fig. 9H, p = 0.0337).

#### Spatial parameters

**Fig. 10:**
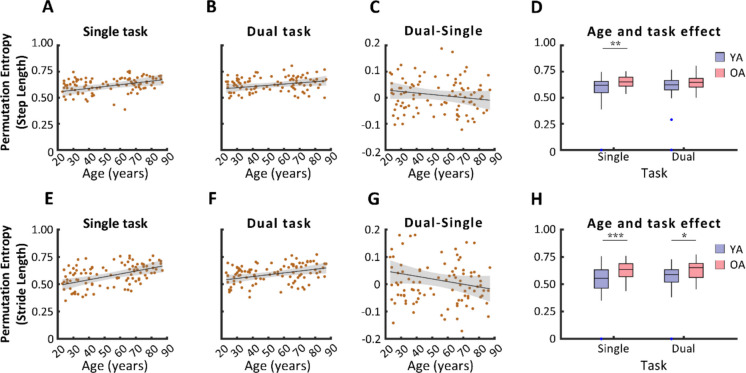
Relationship between age and permutation entropy (PE) of spatial gait parameters during single- and dual-task walking. Panels **A**–**D** show results for step length, **E**–**H** for stride length, and **I**–**L** for stride width. Black trend lines with gray shaded areas indicate the fitted regression and its 95% confidence interval. Box-and-whisker plots display data for younger (*n* = 60) and older (*n* = 41) participants, shown in light blue and light red, respectively. Blue and red dots mark statistical outliers. **A** PE for step length during single-task walking significantly increases with age (Spearman’s *p* < 0.05). **B** PE for step length during dual-task walking as a function of age. **C** Difference in PE between single- and dual-task conditions as a function of age. **D** Combined effects of age group and task condition on PE for step length (**p < 0.01, age effect; Mann–Whitney test). **E** PE for stride length during single-task walking significantly increases with age (Spearman’s *p* < 0.05). **F** PE for stride length during dual-task walking significantly increases with age (Spearman’s *p* < 0.05). **G** Difference in PE between single- and dual-task conditions as a function of age. **H** Combined effects of age group and task condition on PE for stride length (*p < 0.05, ***p < 0.001, age effect; Mann–Whitney test)

Analysis of step length PE revealed significant positive correlation with age (Fig. 10A, *ρ* = 0.318, *p* = 0.0013) and difference between OA and YA (Fig. 10D, p = 0.0017) during single task condition. However, the relationship between stride length PE and age was significant for both task conditions (Fig. 10E, *ρ* = 0.405, *p* < 0.0001 and Fig. 10F, *ρ* = 0.299, *p* = 0.0026). PE of stride length was elevated in OA compared to YA in single and dual task conditions (Fig. 10H, p = 0.0004 and *p* = 0.0113, respectively). There was no difference between single and dual task condition for PE of any spatial gait parameters.

## Discussion

Our findings demonstrate that aging is accompanied by progressive increases in both the magnitude (MAD, CoV) and the spatiotemporal orderliness (PE) of gait fluctuations, particularly under cognitive load. This pattern suggests a dual contribution of age-related decline in neuromotor precision and compensatory recruitment of higher-order control processes to maintain locomotor stability. The accentuation of these effects during dual-task walking underscores the vulnerability of cognitive–motor integration with aging, supporting the concept that gait variability and orderliness are sensitive indicators of early functional decline and reduced resilience of the motor control system.

These results align with earlier studies demonstrating that gait variability increases with age and that dual-tasking amplifies stride-to-stride fluctuations, reflecting impaired cognitive–motor coordination [29, 39–41, 59]. Previous work has also shown that entropy-based metrics can capture nonlinear aspects of gait control, which tend to decrease in neurodegenerative and cognitively impaired populations [52, 53]. Our data extend these observations by demonstrating that permutation entropy—a measure of the temporal complexity of gait—increases with age in a cohort of community-dwelling adults, suggesting that the aging motor system may adopt more irregular but adaptable gait patterns to preserve stability under cognitive load.

What is novel in the present study is the integrated application of robust (MAD) and relative (CoV) dispersion measures together with nonlinear complexity analysis (PE) on a comprehensive set of gait parameters capturing different aspects of locomotion within the same experimental framework. This multidimensional approach enables simultaneous evaluation of the magnitude and organization of gait fluctuations, offering a more detailed characterization of neuromotor adaptability with aging. Furthermore, by embedding this methodological framework within the translational context of the Semmelweis Study, this work establishes a bridge between detailed gait analytics and large-scale preventive aging research—an approach that is unique among current studies of gait variability and aging. Finally, our findings provide a rationale for selecting gait measures that capture variability and complexity based on their sensitivity to aging, thereby optimizing the gait analysis protocol of the Semmelweis Study and enabling the formulation of targeted, mechanism-driven hypotheses.

Gait is increasingly recognized as an integrative neurobehavioral function that depends on the coordinated activity of cortical, subcortical, and spinal circuits, as well as intact sensory feedback and vascular integrity [21–26]. The observed increase in gait variability with age likely reflects cumulative microstructural and functional alterations across these systems [27]. Age-related degradation of white matter tracts [60], particularly within the fronto-striatal and corticospinal pathways, impairs the efficiency of motor signal transmission and sensorimotor integration, leading to less predictable and more irregular step timing. In parallel, reduced cerebral perfusion and neurovascular function—hallmarks of vascular aging—may contribute to impaired cortical control of gait [60, 61], particularly under conditions of increased cognitive load [62].

While it is known that gait variability increases with age [38, 39, 63–65], the present study provides a comprehensive comparison of gait variability metrics by simultaneously applying two complementary measures—MAD and CoV. MAD is a robust indicator of stride-to-stride dispersion that is less affected by extreme values. In contrast, CoV is more sensitive to outliers. We observed no significant right–left asymmetry in gait variability, indicating that the right-leg data reliably capture both age- and task-related effects.

For temporal gait parameters, MAD proved more sensitive to dual-task effects, whereas CoV more effectively detected age-related differences. Regarding spatial parameters such as step length and stride length, CoV showed superior performance in identifying both age- and task-dependent changes. MAD was also able to reveal the dependence of stride width variability on age and task condition. For gait cycle parameters, MAD and CoV performed comparably in detecting both aging and task effects. Finally, among balance-related metrics, CoV generally demonstrated greater sensitivity to age and task influences than MAD Among gait variability parameters, our results indicate that variability in spatial gait measures represents the most sensitive marker of aging, with effects that are further accentuated under dual-task conditions.

Various analytical approaches have been applied in gait research to characterize temporal complexity and orderliness [52, 66]. Prior work using approximate entropy and sample entropy [65, 67] revealed age-associated alterations in gait dynamics. Permutation entropy (PE), used here due to its applicability to short gait sequences [53, 54], demonstrated the strongest association with age compared to MAD and CoV. This suggests that aging is accompanied by a pronounced reduction in spatiotemporal orderliness, exceeding the magnitude of change observed in traditional variability measures. Notably, PE was relatively insensitive to task effects making it a promising biomarker of gait aging that can be assessed without a dual-task paradigm.

The observed age-related increase in PE of temporal and spatial gait parameters indicates a less ordered gait pattern in older adults. Although this appears counterintuitive in light of the *decomplexification theory* of aging—which posits a progressive decline in physiological complexity [68, 69]—the findings may reflect compensatory adaptations in neural control rather than mere degradation [54, 70]. Elevated PE could represent an adaptive mechanism wherein additional or alternative neural networks are recruited to maintain gait stability as efficiency declines [70, 71]. This interpretation aligns with the broader concept of neurobiological compensation in aging, where increased neural engagement supports preserved performance across motor and cognitive domains [69]. Future longitudinal analyses will be critical to distinguish compensatory from maladaptive increases in PE. We hypothesize that adaptive increases in PE will be associated with preserved cognitive and functional performance, whereas maladaptive increases will predict accelerated decline, instability, and adverse outcomes. Tracking PE trajectories in conjunction with vascular and cognitive markers will enable differentiation between these patterns.

The dual-task paradigm provides a sensitive probe of the interplay between executive function and gait control [72, 73]. The disproportionate increases in gait variability and complexity observed during dual-task walking in older adults highlight a reduced ability to allocate attention between cognitive and motor demands [29, 52, 59, 74–77]. This finding supports the “cognitive–motor interference” model, in which the shared neural resources underlying executive control and locomotion become increasingly taxed with age [78]. Declines in attentional flexibility, working memory, and inhibitory control may thus manifest as instability and irregularity in gait when attention is divided [79, 80].

Notably, the combined use of MAD, CoV, and PE revealed complementary aspects of this phenomenon. Whereas MAD and CoV quantified the amplitude and ratio of gait fluctuations, PE captured alterations in the temporal structure of gait cycles—reflecting diminished regularity and predictability under cognitive stress. Together, these measures offer a multidimensional profile of gait control that integrates both motor variability and neural adaptability.

The present findings have important implications for large-scale population studies such as the Semmelweis Study, which aims to identify early functional markers of unhealthy and accelerated brain aging within the Hungarian population [19]. The metrics applied in this pilot—MAD, CoV, and PE – represent key components of the Semmelweis Study gait analysis pipeline, which has been developed to provide a multidimensional characterization of locomotor control and its alterations with age and cognitive load [20]. In the main study, these measures will be complemented by analyses of gait asymmetry, traditional spatiotemporal gait parameters (e.g., stride length, cadence), and their task-dependent modulation under dual-task conditions. Beyond examining individual metrics, the Semmelweis Study will employ advanced analytical frameworks to identify patterns of co-occurrence between gait alterations and cognitive changes, revealing how declines in executive function, attention, and memory parallel or precede disruptions in locomotor control [19]. This integrative approach aims to distinguish signatures of healthy, adaptive aging from those indicative of accelerated or pathological brain aging. Gait variability and entropy-based measures thus offer promising, non-invasive indicators of subtle neuromotor and cognitive decline, and their integration into longitudinal protocols may enable the detection of early deviations in functional aging trajectories – well before overt symptoms emerge. Importantly, these metrics may be particularly sensitive to accelerated cerebrovascular and brain aging processes, which are among the most critical determinants of unhealthy aging in Central and Eastern Europe [19]. Subtle impairments in cerebral blood flow regulation, neurovascular coupling, and endothelial function [61] can alter the neural control of gait long before structural brain changes or cognitive deficits become clinically apparent. Therefore, quantitative gait analysis may serve as an early behavioral proxy for cerebrovascular health, complementing direct physiological assessments such as neurovascular coupling responses, endothelial function, microvascular perfusion, and sensitive computerized cognitive assessments. While the present results highlight the sensitivity of specific metrics—particularly spatial variability and temporal complexity—we anticipate that initial phases of the Semmelweis Study will retain a multidimensional panel of gait measures. This approach will allow data-driven identification of the most informative subset of metrics for large-scale deployment. In the Semmelweis Study, these gait metrics will be integrated with vascular and cognitive data using multivariate analytical approaches, including mediation analyses to assess vascular contributions to gait–cognition relationships, clustering methods to identify multimodal phenotypes, and risk stratification models to predict adverse aging trajectories. The Semmelweis Study—by design—captures a socioeconomically and occupationally diverse population that reflects the health risk profile characteristic of Hungary, including high prevalence of vascular risk factors, obesity, and metabolic syndrome [19]. Within this context, sophisticated gait analysis can provide a sensitive, integrative marker of unhealthy and accelerated brain aging, facilitating the identification of modifiable determinants at a stage when preventive intervention remains possible. This aligns with the broader translational mission of the program: to uncover early functional biomarkers that inform public health strategies and workplace-based prevention.

The study operates in parallel with the Semmelweis–EUniWell Workplace Health Promotion Model Program, which develops and tests interventions targeting the risk factors identified through the Semmelweis Study. These initiatives are coordinated under the umbrella of the Fodor József Center for Prevention and Healthy Aging, established by Semmelweis University to advance research on the causes of unhealthy aging and to develop evidence-based preventive measures. Together, these programs form the institutional foundation of a comprehensive, translational strategy to understand, prevent, and mitigate unhealthy aging in the Hungarian population. By integrating sensitive gait metrics with multimodal assessments of vascular, metabolic, and cognitive health, this framework provides a unique opportunity to evaluate the effectiveness of preventive interventions aimed at extending healthspan and preserving brain resilience across the lifespan.

While this pilot study provides valuable insight into the multidimensional characterization of gait variability, several limitations should be acknowledged. The present cohort represents relatively healthy, community-dwelling adults, and may under-represent frail or institutionalized populations. In more vulnerable groups, gait variability is expected to increase further, whereas permutation entropy may follow a non-linear trajectory, potentially decreasing in advanced pathological states due to loss of adaptive complexity. The cross-sectional design of this pilot study precludes causal inference, and longitudinal follow-up is required to determine whether increased variability and entropy predict future decline in cognitive or physical performance. The sample size, though sufficient for detecting age effects, limits the generalizability of subgroup analyses. Future studies will be guided by the preliminary evidence presented in this paper and will expand upon this pilot by incorporating neuroimaging, vascular biomarkers, and cognitive testing within the Semmelweis Study cohort. Such integrative analyses will clarify the mechanistic pathways linking vascular and neural aging to gait control and may inform early interventions targeting resilience and healthy mobility in aging populations.

In conclusion, this study demonstrates that aging and cognitive load are associated with increased magnitude and complexity of gait variability, reflecting reduced neuromotor precision and altered cognitive–motor integration. These multidimensional gait metrics—particularly when combined—offer sensitive indicators of early functional decline and represent highly informative components within the Semmelweis Study’s biomarker framework. Together with vascular, metabolic, and cognitive assessments, they are expected to contribute substantially to identifying early determinants of unhealthy brain aging and to guiding preventive strategies aimed at promoting healthy longevity.

## Data Availability

The datasets generated and analyzed during the present study are not publicly available due to privacy considerations. However, anonymized data may be obtained from the corresponding author upon reasonable request and will be provided in compliance with institutional and ethical regulations.
